# A School-Based Study of Irritable Bowel Syndrome in Medical Students in Beijing, China: Prevalence and Some Related Factors

**DOI:** 10.1155/2014/124261

**Published:** 2014-10-13

**Authors:** Yang Liu, Liang Liu, Yi Yang, Yuxi He, Yanli Zhang, Miao Wang, Shuo Chen, Shukun Yao

**Affiliations:** ^1^Gastroenterology Department, China-Japan Friendship Hospital, Beijing 100029, China; ^2^Beijing University of Chinese Medicine, Beijing 100029, China; ^3^Graduate School of Peking Union Medical College, Chinese Academy of Medical Sciences, Beijing 100073, China

## Abstract

*Purpose*. To investigate the prevalence and some related factors about irritable bowel syndrome (IBS) in medical students. *Methods*. A cross-sectional study was carried out from February 2014 to Jun 2014 in Beijing University of Chinese Medicine, Beijing, China. All participants were asked to completed self-administered questionnaires. *Results. *Seven hundred and sixty-seven medical students (23.26 ± 2.88 years, 25.6% males) completed the survey. The prevalence of IBS was 33.3%, with a high prevalence in women (36.1%). Among the IBS patients, 112 cases were IBS-M (43.9%) and 77.6% had moderately severe IBS. There were no statistical differences between control group and IBS patients in anxiety and depression scores (*P* > 0.05). The total score of Pittsburgh sleep quality index (PSQI) was significantly higher for medical students with IBS and 35.5% of IBS patients had severe sleep disorder; the scores of child trauma questionnaire (CTQ) and student-life stress inventory (SLSI) were also higher in IBS patients. Sex and sleep disorder were independently associated with IBS (OR, 1.914, 95%CI, 1.281–2.860; OR, 1.143, 95%CI, 1.074–1.216). *Conclusion*. Our study has many valuable findings and they may provide valuable suggestions for the necessary intervention and treatment measures towards medical students.

## 1. Introduction

Irritable bowel syndrome (IBS) is a functional gastrointestinal disorder, characterized by abdominal pain or discomfort in association with altered bowel habits [1]. IBS is common in the general population; the prevalence of this disease in European countries was about 20%; in recent years, the morbidity of Asian countries is rising year by year, which is near to that in Western countries [2–5]. Without any biological markers, the diagnosis of IBS mainly depends on the patient's symptoms. According to Rome III criteria, IBS patients were subdivided into diarrhea-predominant IBS (IBS-D), constipation-predominant IBS (IBS-C), mixed type IBS (IBS-M), and unsubtyped IBS (IBS-U) [6].

Although the pathophysiology of IBS is unclear, gender, diet, lifestyle, psychiatric disorders, sleep problems, early adverse life events (EALs), and stress have been considered as risk factors to IBS, but they are controversial [3–5, 7–15]. Medical students are a special group, characterized by tremendous cognitive and emotional changes. At the same time, due to massive study and exams load, increasing fierce competition for jobs, undesirable living, and eating habits, medical students may be an especially high-risk population for IBS [3]. In recent years, the number of IBS research papers, relating to medical students, has increased in China and the rest of the world [3–5, 16]; however, to the best of our knowledge, no comprehensive survey (including prevalence, diet, lifestyle, psychiatric disorders, sleep problems, EALs, and stress) about IBS using Rome III criteria has been carried out on medical students in Beijing, China, nowadays. The information about prevalence and related factors in medical students may provide targeted recommendations for the treatment of IBS, which can effectively improve the clinical symptoms of IBS patients and enhance their learning efficiency and quality of life.

So the aims of this study were (1) to investigate the prevalence of IBS in medical students according to Rome III criteria and the related factors with IBS and (2) to evaluate the risk factors associated with the disorder.

## 2. Materials and Methods

### 2.1. Study Setting and Ethics Statement

This cross-sectional study was carried out from February 2014 to June 2014 in Beijing University of Chinese Medicine, Beijing, China. All participants gave their written informed consent prior to completing self-administered questionnaires. The study protocol was approved by the ethics committee of the China-Japan friendship hospital, Beijing, China.

### 2.2. Participants

This study included medical students from the first to the seventh year, and we employed a multistage stratified random sample method [4, 8]. Using the following formula, *n* = *pq*/(*d*/*t*)^2^ = *t* ^2^ × *pq*/*d* ^2^, where *t* = 1.96 (error of the first kind), *p* = 20% (estimated prevalence), *q* = 1 − *p*, and *d* = 15% × *p* (permissive error), the expected minimal sample size for this study was 683. According to the 90% response rate, the number of students should be 759. To ensure reliability, we proceeded to recruit 843 medical students into the study.

### 2.3. Criteria of Exclusion

Subjects with self-reported organic gastrointestinal disorder, family history of cancer, weight loss, anemia, bloody stools, abnormal laboratory findings, other alarming signs, and those who had previously undergone gastrointestinal surgery were excluded from our study [1].

### 2.4. Questionnaires

#### 2.4.1. Personal General Information, Lifestyle, and Eating Habits

Personal data, such as sex, age, height, weight, and lifestyle and eating habits, such as exercise frequency, smoking, and alcohol consumption, were included in the questionnaire. With the grade, low means the students from the first to the fourth year, and high means from the fifth to the seventh year.

#### 2.4.2. Chinese Version of Rome III Criteria for Diagnosis of IBS

The Chinese version of Rome III criteria for diagnosis of IBS has been widely used [1, 17, 18]. The diagnosis of IBS was based on the presence of abdominal pain or discomfort which had to occur at least 3 days per month for at least three months during the previous six months, with at least two or more of the following conditions: symptoms associated with a change in frequency or form of stool and pain improved after defecation. Patients with IBS were divided into diarrhea-predominant IBS (IBS-D), constipation-predominant IBS (IBS-C), mixed IBS (IBS-M), and unsubtyped IBS (IBS-U) according to the proportion of hard and lumpy stools.

#### 2.4.3. “Severity of IBS” Questionnaire

The IBS severity questionnaire includes five items: the severity of perceived pain, the presence and severity of abdominal distension, the frequency of abdominal pain or discomfort, satisfaction with bowel habits, and the quantification of interference in the patient's general lifestyle by these symptoms. Each item has a 100-point scale from 20 to 100 score and the total severity score is the sum of the five items. Patients were classified as having mild IBS (IBS severity score 75–175), moderately severe IBS (score 175–300), and severe IBS (score > 300) [19, 20].

#### 2.4.4. Hospital Anxiety and Depression Scale (HADS)

HADS was widely used in China [17, 18]. It has 14 questions (with two 7-item subscales) and was used in estimating the emotional disorders of anxiety and depression status in patients. For each subscale, the result fell into 3 grades as follows: normal cases (0–7), borderline cases (8–10), and severe cases (over 11) [17, 18].

#### 2.4.5. Pittsburgh Sleep Quality Index (PSQI)

PSQI is a 19-item, self-administered questionnaire [21]. It has been widely used to assess the sleep quality of subjects [8]. The contents of the PSQI include seven items: subjective sleep quality, sleep latency, sleep duration, habitual sleep efficiency, sleep disturbances, use of sleep inducers, and daytime dysfunction. Each item uses a three-point response scale, the total score was used to calculate the PSQI global index (ranging from 0 to 21); a total score > 5 means poor sleepers, and when the total score is up to or greater than 8, it means a severe sleep disorder.

#### 2.4.6. Child Trauma Questionnaire (CTQ)

CTQ, which has 28 items, was developed by Bernstein et al. [22]. CTQ consists of five subscales (emotional abuse, physical abuse, sexual abuse, emotional neglect, and physical neglect, each subscale has five items) to evaluate the degree of child trauma (one type of EALs) and three items for validity evaluation. Each item has a 5-point scale from 1 to 5 score; the total score ranges from 25 to 125 [23].

#### 2.4.7. Student-Life Stress Inventory (SLSI)

SLSI was designed by Gadzella [24]. SLSI has 51 items with five stress categories (frustration, conflicts, pressures, changes, and self-imposed) and four categories of reactions to stressors. Each item uses a 5-point scale; the total score is the sum of the nine categories. The purpose of the SLSI was to assess the mean responses to different stressors and reactions to stressors among students [24].

### 2.5. Statistical Analysis

Statistical analysis was performed using the SPSS version 19.0 (IBM Corporation, NY, USA). Categorical variables were analyzed by Pearson's *χ* ^2^ or Fisher's exact test. The *t*-test was used to compare group differences for continuous variables; data is presented as mean ± SD. Logistic regression analysis was used to assess the possible risk factors. The odds ratio (OR) with a 95% confidence interval (CI) was calculated. All calculated *P* values were two-tailed and *P* < 0.05 was considered statistically significant.

## 3. Results

### 3.1. Response Rate, Overall Characteristics, Lifestyle, and Eating Factors of Participants

Of the 843 medical students, 767 (91.0%) completed the survey, and they were included in the final analyses. The age of the medical students ranged from 18 to 27 years, with a mean age of 23.26 ± 2.88 years; 196 (25.6%) were males and 571 (74.4%) were females. The demographic characteristics, lifestyle, and eating factors of participants are listed in Tables 1 and 2. It is apparent from Table 2 that significant difference was found between IBS and control groups in seafood diet; IBS patients had a lower frequency in this item.

### 3.2. Prevalence of IBS

Of the 767 medical students, 255 fulfilled the criteria for having IBS, the prevalence of IBS was 33.3%, for females, the prevalence was 36.1% (206/571), and for males, 25.0% (49/196) of the cases were found to be positive. At the same time, we found that, for women, the students with low grade had a higher prevalence of IBS (45.9%, 67/146), and the high grade was 32.8% (139/425) (*P* = 0.015); for men, no statistical difference was found (23.1%, 24/104 versus 27.2%, 25/92 *P* = 0.514). Among the IBS patients, 112 cases were IBS-M (43.9%), 79 cases were IBS-D (31.0%), 49 cases were IBS-U (19.2%), and 15 cases were IBS-C (5.9%). Female predominance was IBS-M (45.1%), and male predominance was IBS-D (44.9%). Of the 255 IBS patients, 20.4% were mild IBS, 77.6% had moderately severe IBS, and 2.0% were severe cases.

### 3.3. HADS and PSQI Scores in IBS Patients and Controls

The anxiety and depression scores in IBS patients were 4.89 ± 2.87 and 4.34 ± 2.77, for control group, there were 4.58 ± 2.95 and 4.23 ± 2.94, and no statistical differences were found (Table 3). However, for females, IBS patients had a higher score than healthy controls in anxiety (4.83 ± 2.67 versus 4.23 ± 2.85, *P* = 0.015).

As shown in Table 3, the total score of the PSQI was significantly higher for medical students with IBS than for those without IBS; on the other hand, for females, the score was 6.65 ± 2.67 in IBS cases and 5.42 ± 2.73 in controls (*P* < 0.001); for males, the scores were 5.96 ± 2.38 and 4.97 ± 2.45 (*P* = 0.014). According to the PSQI score standard, the prevalence of severe sleep disorder was 35.5% in IBS patients and 17.8% in subjects without IBS.

### 3.4. CTQ and SLSI Scores in IBS and Control Groups

Table 4 illustrates that the score of emotional neglect was much higher in IBS patients (*P* = 0.045); the other 4 subscales and the total score of CTQ were also higher in IBS group, but there were no significant differences.

For SLSI, the total score of SLSI and the other seven categories showed statistical differences between IBS and control groups (Table 5). Similar results were obtained in females (Table 6); however, for males, no differences were found in the total score of SLSI and nine categories between IBS patients and health group.

### 3.5. Risk Factors for IBS

After the univariate analysis, to find independent risk factors for IBS, multivariable logistic regression analysis was performed. Sex and sleep disorder were independently associated with IBS (Table 7).

Table 7 shows the evaluation of risk factors for irritable bowel syndrome by multivariate logistic regression analysis.

## 4. Discussion

To our knowledge, this was the first comprehensive survey using the Rome III criteria to assess the prevalence of some related factors in medical students in Beijing, China. In this study, the prevalence of IBS in medical students was 33.3%, 43.9% of those with IBS had IBS-M, and the majority of the patients (77.6%) had moderately severe IBS. IBS patients had a significantly higher PSQI score together with a 35.5% prevalence of severe sleep disorder. In the assessments of CTQ and SLSI, the scores in patients were also higher.

Our survey suggests that medical students have an even higher prevalence of IBS, more female students suffered from IBS, and gender may be a risk factor of this disease. The results are in line with that in previous studies [3, 4]. Considering the special living environment (countless examinations and heavy clinical practice in hospital) together with lacking the ability of self-adjustment and adaptation, medical students may be an especially high-risk population for IBS. At the same time, we found that female students had a higher prevalence than male students, speculating that the difference of hormone levels may play a role [25]. In addition, women are more likely to have mental problems and sleep disorders, which may be related to the high prevalence of IBS. For females, the students with low grade had a higher prevalence of IBS, which is contrary to the previous study [4]. Differences in study groups and evaluation standard may explain the relative lacking of medical knowledge; low health care seeking rate may also give rise to the high prevalence of IBS.

Unfortunately, in the survey about eating habits and lifestyles, we only found significant difference in seafood diet between IBS and control groups; IBS patients had a lower frequency in this item. It may be true that some IBS patients had food intolerance. Gastrointestinal symptoms appeared after eating seafood, which made them artificially eliminate the seafood diet later.

In our survey, no statistical differences were found between IBS patients and controls in anxiety and depression scores. However, in the subgroup analysis, female patients had obviously higher score in anxiety score, which is similar to a recent study from Pakistan [3]. Studies have shown that, compared to healthy controls, 40%–60% of IBS patients with obvious mental abnormalities and early relieving mental disorders might be useful for an effective management of IBS [10, 26]. Psychosocial factors can lead to dysfunction of the brain-gut axis, which in turn causes dysfunction of the gut through neural, neuroimmune, and neuroendocrine pathways [27].

To date, no study has been conducted to explore the association between IBS and sleep disorder in medical students in China. From our data, the total score of the PSQI was significantly higher for medical students with IBS than for those without IBS, and the prevalence of severe sleep disorder was 35.5% in IBS group. Sleep disorder was independently associated with IBS. A study from China found that 34.3% of the middle school students had sleep problems, and poor sleep was independently associated with IBS [8]. It has been demonstrated that poor sleep is a dangerous stress factor, which can seriously influence the cognition, emotion, somatic reaction, and gastrointestinal function [28]. A population-based study showed that sleep disorder was associated with increased odds for multiple gastrointestinal symptoms [29]. Studies have shown that poor sleep can change the intestinal sensitivity, which increases the patient's perception of clinical symptoms [29, 30]. Disruption of the normal biological rhythm may lead to alteration in visceral motility and sensitivity, thus changing the colonic physiological function of the gut.

Epidemiologic studies have found an increased prevalence of EALs in IBS patients [14, 31].

In our study, we have shown that the score of emotional neglect was much higher in IBS patients.

It is the first research using the CTQ questionnaire to investigate the relationships between EALs and IBS in medical students. In USA, more than 60% adults reported that they had at least one EAL before the age of 18 [32]. EALs may cause the disturbance of central nerve system including hypothalamic-pituitary-adrenal (HPA) axis, cognitive behavior, autonomic responsiveness, and emotional regulation, thus participating in the occurrence and development of IBS [14]. The other 4 subscales and the total score of CTQ were also higher in IBS group, but there were no significant differences. We speculate that, with expansion of the sample size, the result may be different.

In the present study, SLSI scores were significantly increased in patients with IBS and this phenomenon is more pronounced in women. However, there were no statistical differences in males. To our knowledge, this was the first investigation using the SLSI to assess the relationships between stress factors and IBS in medical students. In recent years, increasing evidence supports the role of stress in the pathophysiology of IBS [13, 15]. The effects of stress on functions of gut are universal, and the major effects of stress on gastrointestinal tract include alteration in gastrointestinal motility and visceral perception, increase in intestinal permeability, changes in intestinal endocrine, negative effect of intestinal mucosal blood flow, and negative effects on intestinal microbiota [13]. Differences in the following factors between women and men may explain why our findings were not the same in gender about the SLSI scores: hormone level, social role and health care seeking behavior, and physiological and psychological reactions together with coping styles when exposed to stressors.

The strength of this research was the high questionnaire responses and enough sample size to ensure the credibility of our findings. Also, we have some limitations. First, the survey was restricted to medical students, which might influence the generalizability of our results. Second, the study was based on self-reporting questionnaires, without using gastrointestinal endoscopy to exclude structural intestinal diseases; sometimes, it is unpractical in a cross-sectional survey with a large sample.

## 5. Conclusion

We conclude from this cross-sectional study that the prevalence of IBS in medical students is 33.3%, the scores of CTQ and SLSI were also higher in IBS patients, and sex and sleep disorder were independently associated with IBS. It may provide valuable suggestions for the necessary intervention and treatment measures towards medical students; too much attention was given just to the intestinal symptoms formerly. Further prospective studies evaluating the definite pathological mechanisms should be carried out.

## Figures and Tables

**Table 1:** Characteristics of medical students with or without IBS *n* (%).

	Total (*n* = 767)	IBS (*n* = 255)	Non-IBS (*n* = 512)	*χ* ^2^/*t*	*P* value
Sex				8.151	0.005
Male	196 (25.6%)	49 (19.2%)	147 (28.8%)		
Female	571 (74.4%)	206 (80.8%)	365 (71.2%)		
Age (yr)	23.26 ± 2.88	22.95 ± 2.87	23.45 ± 2.87	−2.267	0.024
Height (m)	1.64 ± 0.07	1.64 ± 0.07	1.65 ± 0.07	−2.975	0.005
Weight (kg)	56.62 ± 11.07	56.16 ± 11.57	56.85 ± 10.82	−0.816	0.459
BMI (kg/m^2^)	20.76 ± 2.90	20.89 ± 3.32	20.69 ± 2.68	0.862	0.389
Nationality				0.086	0.881
Han	713 (93.0%)	238 (93.3%)	475 (92.8%)		
Minority	54 (7.0%)	17 (6.7%)	37 (7.2%)		
Grade				1.617	0.220
Low	251 (32.6%)	91 (35.7%)	160 (31.1%)		
High	516 (67.4%)	164 (64.3%)	352 (68.9%)		

IBS: irritable bowel syndrome.

**Table 2:** Lifestyle and eating habits of medical students with or without IBS *n* (%).

	Total (*n* = 767)	IBS (*n* = 255)	Non-IBS (*n* = 512)	*χ* ^2^	*P* value
Smoking				0.130	0.790
No	750 (97.9%)	249 (97.6%)	502 (98.0%)		
Yes	17 (2.1%)	6 (2.4%)	10 (2.0%)		
Alcohol consumption				2.201	0.333
No	721 (94.1%)	244 (95.7%)	478 (93.2%)		
Yes	45 (5.9%)	11 (4.3%)	34 (6.7%)		
Exercise frequency				2.280	0.516
<1 time/week	258 (33.7%)	79 (31.0%)	179 (35.0%)		
1 or 2 times/week	289 (37.7%)	104 (40.8%)	185 (36.2%)		
≥3 times/week	220 (28.6%)	72 (28.2%)	148 (28.8%)		
Coffee				2.504	0.475
Seldom	713 (93.0%)	236 (92.9%)	476 (93.0%)		
Often	54 (7.0%)	19 (7.1%)	36 (7.0%)		
Carbonated beverage				2.556	0.279
Seldom	618 (80.7%)	209 (82.0%)	409 (80.0%)		
Often	149 (19.3%)	46 (18.0%)	103 (20.0%)		
Eating fruits				1.072	0.585
Seldom	184 (24.0%)	58 (22.7%)	126 (24.7%)		
2–6 times/week	409 (53.4%)	134 (52.5%)	275 (53.8%)		
Almost every day	174 (22.6%)	63 (24.7%)	111 (21.5%)		
Pure milk drinking				0.206	0.902
Seldom	429 (56.0%)	143 (56.1%)	286 (56.0%)		
2–6 times/week	266 (34.7%)	90 (35.3%)	176 (34.4%)		
Almost every day	72 (9.3%)	22 (8.6%)	50 (9.6%)		
Acidophilus milk				1.751	0.417
Seldom	354 (46.2%)	113 (44.3%)	241 (47.2%)		
2–6 times/week	371 (48.4%)	131 (51.4%)	240 (47.0%)		
Almost every day	42 (5.4%)	11 (4.3%)	31 (5.8%)		
Spicy food				1.518	0.468
Seldom	248 (32.4%)	76 (29.8%)	172 (33.7%)		
2–6 times/week	421 (55.0%)	148 (58.0%)	273 (53.4%)		
Almost every day	98 (12.7%)	31 (12.2%)	67 (12.9%)		
Greasy food				2.661	0.447
Seldom	299 (39.0%)	96 (37.6%)	203 (39.7%)		
2–6 times/week	366 (51.7%)	132 (51.8%)	264 (51.7%)		
Almost every day	72 (9.3%)	27 (10.6%)	45 (8.6%)		
Meat (pig, cattle, or sheep)				1.688	0.430
Seldom	132 (17.2%)	39 (15.3%)	93 (18.2%)		
2–6 times/week	459 (59.9%)	152 (59.6%)	307 (60.1%)		
Almost every day	176 (22.9%)	64 (25.1%)	112 (21.7%)		
Fish				2.807	0.246
Seldom	470 (61.4%)	167 (65.5%)	303 (59.3%)		
2–6 times/week	287 (37.5%)	85 (33.3%)	202 (39.5%)		
Almost every day	10 (1.2%)	3 (1.2%)	7 (1.2%)		
Seafood diet				11.097	0.025
Seldom	681 (88.9%)	236 (92.5%)	445 (87.1%)		
2–6 times/week	81 (10.6%)	17 (6.7%)	64 (12.5%)		
Almost every day	5 (0.5%)	2 (0.8%)	2 (0.4%)		
Salty food				1.700	0.194
Not preferred	381 (49.6%)	118 (46.3%)	263 (51.3%)		
Preferred	386 (50.4%)	137 (53.7%)	249 (48.7%)		
The days of regular diet				3.295	0.348
6-7 days/week	427 (55.5%)	134 (52.5%)	291 (56.9%)		
3–5 days/week	298 (38.9%)	106 (41.6%)	192 (37.6%)		
≤2 days/week	42 (5.6%)	15 (5.9%)	28 (5.5%)		

IBS: irritable bowel syndrome.

**Table 3:** Comparing mean scores of the HADS and PSQI between IBS and non-IBS.

	IBS	Non-IBS	*t*	*P* value
HADS				
Anxiety	4.89 ± 2.87	4.58 ± 2.95	1.382	0.167
Depression	4.34 ± 2.77	4.23 ± 2.94	0.510	0.610
PSQI (total)	6.52 ± 2.70	5.29 ± 2.65	6.015	<0.001

HADS: hospital anxiety and depression scale; PSQI: Pittsburgh sleep quality index; IBS: irritable bowel syndrome.

**Table 4:** The CTQ scores between IBS and non-IBS.

	IBS	Non-IBS	*t*	*P* value
Emotional abuse	6.09 ± 2.02	5.88 ± 1.56	1.586	0.113
Physical abuse	5.30 ± 1.09	5.21 ± 0.98	1.118	0.264
Sexual abuse	5.22 ± 0.81	5.19 ± 0.89	0.337	0.736
Emotional neglect	7.22 ± 3.41	6.75 ± 2.87	2.004	0.045
Physical neglect	6.07 ± 1.65	6.01 ± 1.75	0.505	0.614
CTQ (total)	29.90 ± 6.58	29.04 ± 6.10	1.781	0.075

CTQ: child trauma questionnaire; IBS: irritable bowel syndrome.

**Table 5:** The SLSI scores between IBS and non-IBS.

	IBS	Non-IBS	*t*	*P* value
(I) Stressors				
Frustration	15.76 ± 3.49	14.38 ± 3.99	2.139	0.033
Conflict	7.00 ± 2.78	6.38 ± 2.41	3.197	0.001
Pressure	11.92 ± 5.48	10.82 ± 3.51	3.349	0.001
Change	7.78 ± 2.63	7.09 ± 2.43	3.598	<0.001
Self-imposed	17.14 ± 4.59	16.79 ± 4.35	1.036	0.301
(II) Reaction to stressors				
Physiological	23.89 ± 6.95	22.45 ± 6.64	2.780	0.006
Emotional	9.76 ± 3.66	8.91 ± 3.29	3.232	0.001
Behavioral	12.49 ± 4.25	11.80 ± 3.96	2.220	0.027
Cognitive	5.10 ± 1.94	5.22 ± 2.29	−0.722	0.471
(III) Totals	110.83 ± 25.93	103.85 ± 21.69	3.936	<0.001

SLSI: student-life stress inventory; IBS: irritable bowel syndrome.

**Table 6:** The SLSI scores between IBS and non-IBS in females.

	IBS	Non-IBS	*t*	*P* value
(I) Stressors				
Frustration	15.89 ± 4.80	14.19 ± 3.91	2.139	0.039
Conflict	6.95 ± 2.73	6.10 ± 2.39	3.197	<0.001
Pressure	12.16 ± 5.81	10.77 ± 3.56	3.349	<0.001
Change	7.86 ± 2.52	7.04 ± 2.45	3.598	<0.001
Self-imposed	17.21 ± 4.63	16.72 ± 4.51	1.036	0.217
(II) Reaction to stressors				
Physiological	24.21 ± 7.21	21.87 ± 6.24	4.058	<0.001
Emotional	9.86 ± 3.69	8.79 ± 3.32	3.582	<0.001
Behavioral	12.28 ± 4.20	11.28 ± 3.70	2.958	0.003
Cognitive	5.25 ± 1.92	4.20 ± 0.30	−0.189	0.850
(III) Totals	111.67 ± 26.76	102.05 ± 21.76	4.657	<0.001

SLSI: student-life stress inventory; IBS: irritable bowel syndrome.

**Table 7:** Evaluation of risk factors for irritable bowel syndrome by multivariate logistic regression analysis.

Variables	*β*	Standard error	Wald	Odds ratio	95% CI		*P* value
					Lower	Upper	
Sex	0.649	0.205	10.026	1.914	1.281	2.860	0.002
PSQI	0.134	0.032	17.934	1.143	1.074	1.216	<0.001

CI: confidence interval.
